# Supplementary figures and images for: A mechanistic modeling framework reveals the key principles underlying tumor metabolism
Source: PLoS Comput Biol. 2022 Feb 11;18(2):e1009841. doi: 10.1371/journal.pcbi.1009841 (PMC8870510; doi:10.1371/journal.pcbi.1009841)

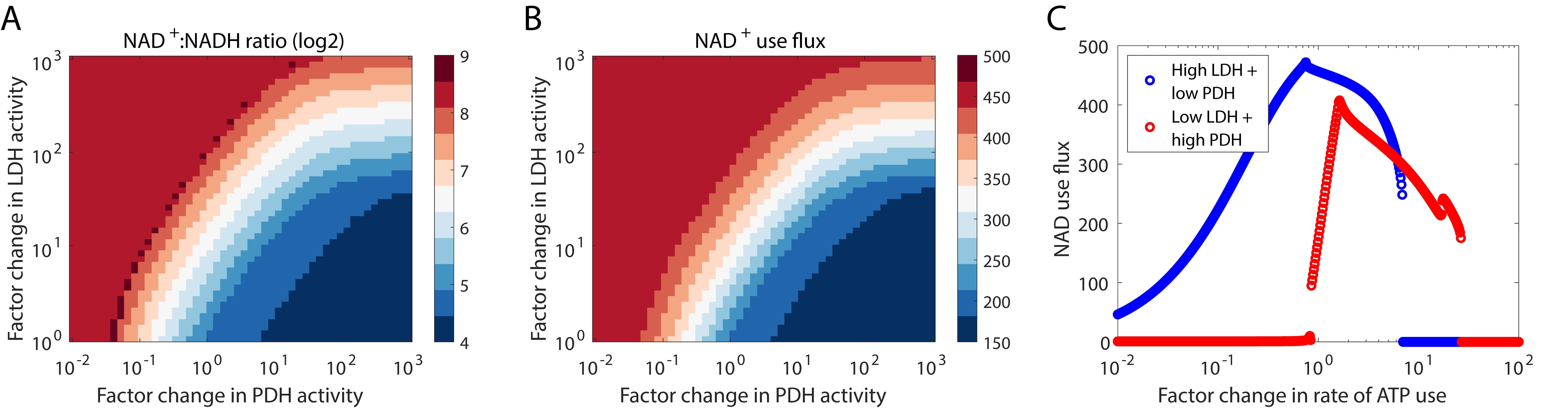

Supplement: S1 Fig — (A) The cytoplasmic NAD+: NADH ratio decreases with an increase in PDH activity. (B) The NAD+ use flux, which determines the proliferation rate when NAD+ is the limiting factor to fast proliferation, decreases with an increase in PDH activity. Note that the Warburg effect phenotype (high LDH, low PDH in the absence of oxygen deprivation) is characterized by a high NAD+ use flux and can thus drive fast proliferation. (C) The regime that maximizes the NAD+ use flux depends on the rate of cellular ATP use. The behavior in panel C is similar to the one shown in Fig 2A: while high LDH, low PDH activity can drive fast proliferation at low ATP consumption rates, high PDH activity is needed to maintain fast proliferation under high ATP use. (TIF) [file pcbi.1009841.s002.tif]

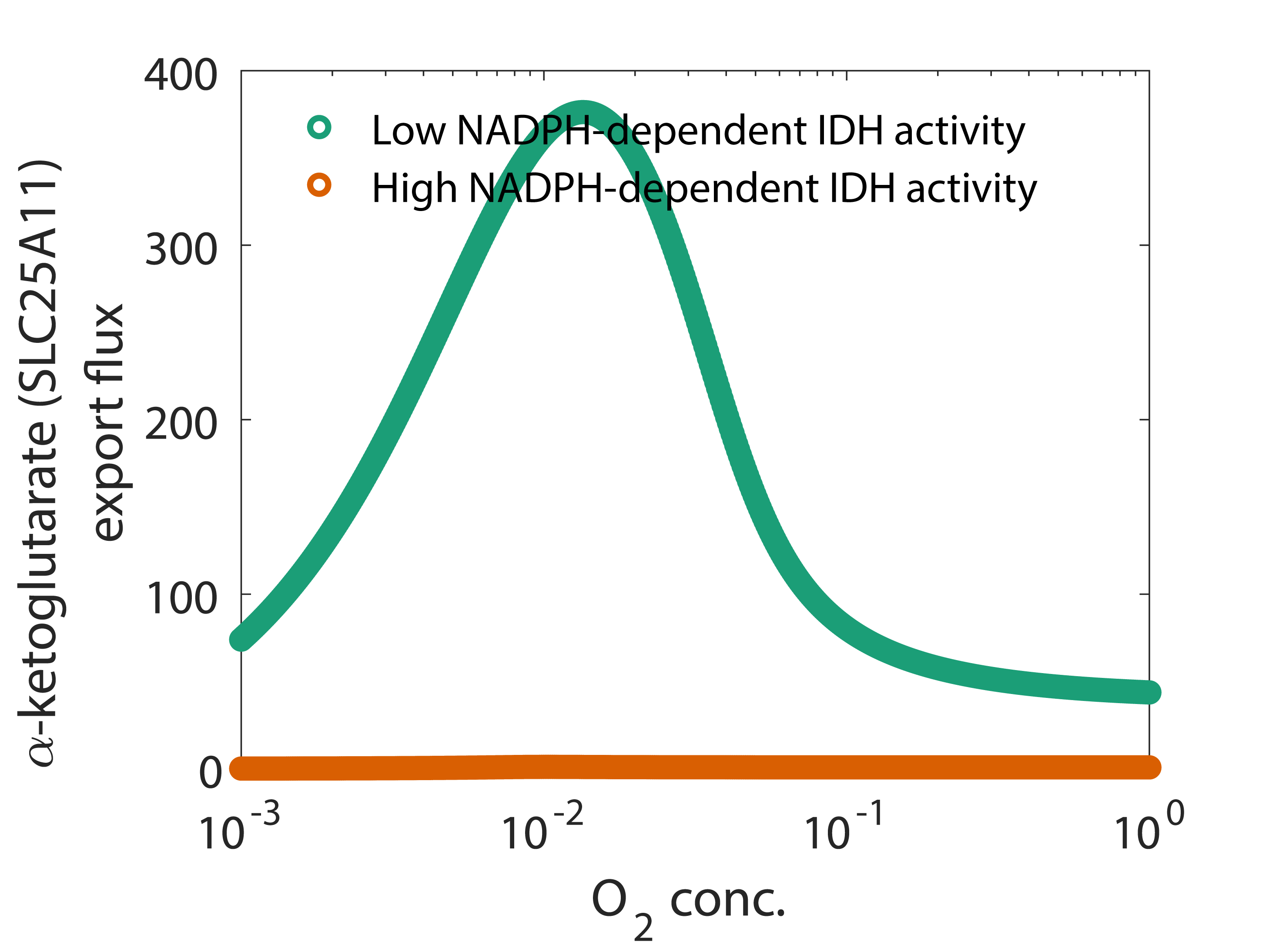

Supplement: S2 Fig — Simulation conditions are the same as shown in Fig 3E in the main text. (TIF) [file pcbi.1009841.s003.tif]
